# Preparation and Gas Sensing Properties of Hair-Based Carbon Sheets

**DOI:** 10.3390/nano12193512

**Published:** 2022-10-08

**Authors:** Zhaofeng Wu, Yidan Xia, Lixiang Liu, Qihua Sun, Jun Sun, Furu Zhong, Min Zhang, Haiming Duan

**Affiliations:** 1 Xinjiang Key Laboratory of Solid State Physics and Devices, Urumqi 830046, China; 2 School of Physics Science and Technology, Xinjiang University, Urumqi 830046, China; 3 School of Physics and Electronic Science, Zunyi Normal College, Zunyi 563006, China

**Keywords:** human hair, biomass carbon material, gas-sensitive properties, carbon sheets

## Abstract

Waste human hair was carbonized into carbon sheets by a simple carbonization method, which was studied as gas sensing materials for the first time. The effect of carbonization temperature on the structure and gas sensing properties of hair-based carbon sheet was studied by scanning electron microscope, X-ray diffraction, infrared spectrum, Raman spectrum, and gas-sensitive tester. The results showed that the carbonization temperature had a significant effect on the structure and gas sensing performance of carbon sheets, which were doped with K, N, P, and S elements during carbonization. However, the sensor of the carbon sheet does not show good selectivity among six target gases. Fortunately, the carbon sheets prepared at different temperatures have different responses to the target gases. The sensor array constructed by the carbon sheets prepared at different temperatures can realize the discriminative detection of a variety of target gases. For the optimized carbon sheet, the theoretical limit of detection of hydrogen peroxide is 0.83 ppm. This work provides a reference for the resource utilization of waste protein and the development of gas sensors.

## 1. Introduction

Carbon materials play an important role in the progress of human civilization. Classic carbon materials such as coal, graphite, and activated carbon have driven social and technological progress [1]. In recent decades, a variety of new carbon materials including fullerenes [2], carbon nanotubes [3], and graphene [4] have had great application prospects in sensing [5], catalysis [6], energy [7], and other fields [8]. However, these new carbon materials are mostly made from non-renewable petrochemical products, such as methane and ethylene [9,10]. In addition, the preparation of these new carbon materials generally requires catalysts, strong acids and strong oxidants, and the preparation process is complex, high-cost, and high environmentally risky [9,10,11]. Therefore, the use of renewable raw materials to develop environmentally friendly, low-cost, easy-to-prepare carbon materials is a major issue to be solved at present. Biomass carbon materials (BCMs) is a material with diverse functions and structures, prepared from renewable biomass in nature through simple carbonization treatment [12]. BCMs are not only energy-saving and environmentally friendly, but also can convert the organic carbon in biomass into solid inorganic carbon, which effectively prevents the organic carbon in biomass from returning to the atmospheric environment in the form of greenhouse gases [13]. BCMs may help to promote carbon neutrality and carbon peak, and achieve the concept of “Using waste to treasure” [14]. What is more, due to the diversity of biomass, its structure and composition are also diverse, which brings a lot of convenience to the preparation and performance regulation of BCMs.

It is well known that doping is a common and effective way to regulate the structure and properties of semiconductors [15,16]. For graphene and carbon nanotubes, it is relatively difficult to regulate their properties by atomic doping. Biomass not only contains C, H, and O elements, but also sometimes contains N element, as well as S, P, K, and other trace elements [17,18]. Therefore, N-doping, or even a variety of co-doping elements, can be realized in the preparation process of BCMs. Yagang Zhang et al. used cellulose carbamate as a precursor to prepare hierarchical N-doped BCMs via simultaneous carbonization and activation. The hierarchical N-doped BCMs have shown excellent performance as an electrode for capacitors and as an adsorption material [19]. Doping is also an important strategy to control the gas sensing properties of semiconductors. Our group carbonized rose tea into K-doped BCMs with the folded structure of dog turbinate. Due to the K-doping, the adsorption capacity of the K-doped BCMs for NH_3_ is effectively increased, and the highly sensitive and anti-interference detection of NH_3_ is realized [20]. Our group also used wool as precursor to prepare N, P, and K co-doped carbon fibers by hydrothermal method. Due to the doping of N, P, and K, as well as abundant functional groups, the carbon fiber can produce different shapes of sensing curves for different target gases. Combined with image recognition technology, multiple target gases can be discriminatively detected only by a single sensor of wool-based carbon fiber [13].

As a common daily waste composed of 51% carbon, 17% nitrogen, 21% oxygen, 6% hydrogen, and 5% sulfur, hairs are easy to obtain [21,22,23,24]. As the carbon content exceeds 50%, hair is a good biomass carbon source. Derya et al. prepared a carbon material with a graphene-like structure using Turk hair as a material and carbonized at 280 °C, and used it in the field of supercapacitors [21]. Haiyan Yan et al. pyrolyzed hairs at temperatures of 800, 900, and 1000 °C to generate highly functional N and S co-doped carbon materials, which was used in the electrodes of lithium-ion batteries and supercapacitors [25]. As far as we know, although BCMs have been used in capacitors, batteries, etc., there have been no reports on hair-based carbon materials as gas-sensitive materials so far. In this study, inspired by the above works, human hairs were used as raw materials to prepare hair-based carbon sheets. As a gas sensing material, the effect of carbonization temperature on the structure and properties of the hair-based carbon sheets was also investigated.

## 2. Materials and Methods

### 2.1. Materials

Hairs were provided by the volunteer from Xinjiang University. Hairs fell off when the volunteer combed their hair every day and the fallen strands were then provided to us for experiments. Ethanol (C_2_H_5_OH (≥99.5%)), formaldehyde (HCHO (37%)), acetone (C_3_H_6_O (99.9%)), and NH_3_ (25%) were purchased from Sinopharm Chemical Reagent Co., Ltd. (Beijing, China). Hydrogen peroxide (H_2_O_2_ (30%)) was purchased from Aladdin Reagent Co., Ltd. (Shanghai, China). All the reagents were analytical reagents.

### 2.2. Sample Preparation

**Figure 1:**
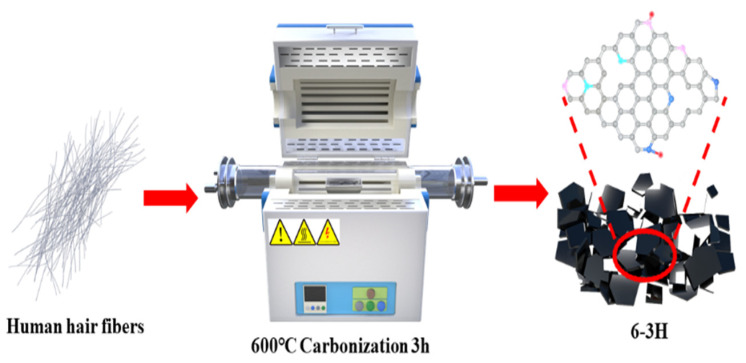
Schematic diagram for the preparation of hair-based carbon sheets.

First, the hairs were washed with deionized water and C_2_H_5_OH. Second, the hairs were dried in a drying oven at 80 °C for 6 h. Third, the dried hairs were cut into short fibers and carbonized at 500, 600, and 700 °C for 3 h under the protection of nitrogen (Figure 1). For the convenience of expression, these samples carbonized at 500, 600, and 700 °C were named 5–3H, 6–3H, and 7–3H. 

### 2.3. Sample Characterization

The morphology, composition and structure were investigated by field emission-scanning electron microscopy (FESEM, S–4800, Hitachi, Tokyo, Japan), mapping (Mapping, S–4800, Hitachi, Tokyo, Japan) and X-ray diffraction (XRD, Bruker D8 Advance, Karlsruhe, Germany). Functional groups of the samples were obtained by Fourier transform infrared spectroscopy (FTIR, Bruker Vertex 70, Karlsruhe, Germany). Raman spectra were recorded using a Raman spectrometer (RAMAN, iHR550, Shanghai, China) at a wavelength of 532 nm.

### 2.4. Preparation and Test of Sensing Chip

The sensing material was mixed with a certain amount of deionized water, ground into a paste. The pastes were uniformly brushed on the interdigital electrode, dried at room temperature for 24 h, and the thickness of the sensing materials was about 320 µm. The length and width of the interdigital electrode sheet were 13 and 7 mm, respectively, and the width between the electrodes was 200 µm. As shown in Figure S1 from the Supplementary Materials, the gas sensing test was performed by a CGS-MT multifunctional detection station at room temperature, which is the same as the previous work [20]. To reduce the interference of the external environment, the temperature and humidity of the testing rooms were controlled at about 25 °C and 33%, respectively. All target gases or vapors were generated by thermal evaporation by the following Equation (1):

Q = (V × C × M)/(22.4 × d × ρ) × 10^−9^ × (273 + T_R_)/(273 + T_C_) (1)

In the above equation, Q and V are the volume of the liquid to be taken and the volume of the test vessel, respectively. M is the molecular weight of the substance, d is the purity of the liquid, C is the concentration of the gas to be dispensed, ρ is the density of the liquid, and T_R_ and T_C_ are the ambient temperature of the test and the temperature inside the test vessel, respectively. The response is defined as follows:

(2) $$\mathrm{Response}=(\frac{I_{G}-I_{R}}{I_{R}})\times 100\%$$

where *I_R_* and *I_G_* are the current of the sensor in the reference gas (air) and target gas (C_2_H_5_OH, HCHO, C_3_H_6_O, NH_3_, and H_2_O_2_), respectively. The response time is defined as the time to reach 90% of the stable response value, and the recovery time is defined as the time to reach within 10% of the initial response value [20].

## 3. Results and Discussion

### 3.1. Morphologies and Microstructures

**Figure 2:**
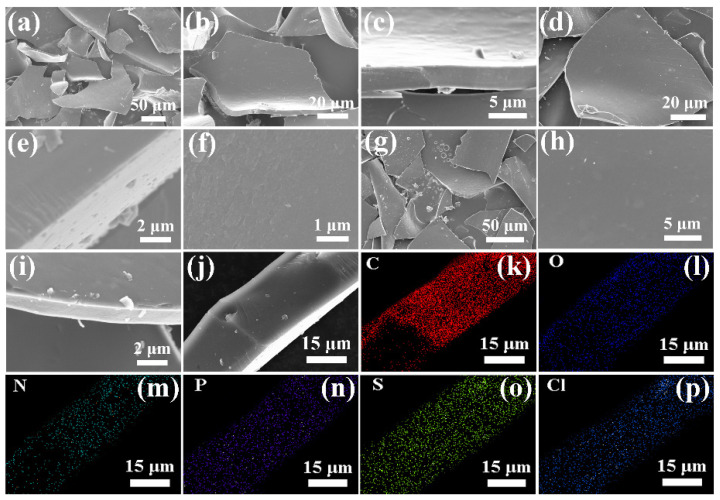
SEM images with different magnification of (**a**–**c**) 5−3H, (**d**–**f**) 6−3H, (**g**–**i**) 7−3H; element mapping of (**j**–**p**) 6−3H.

**Figure 3:**
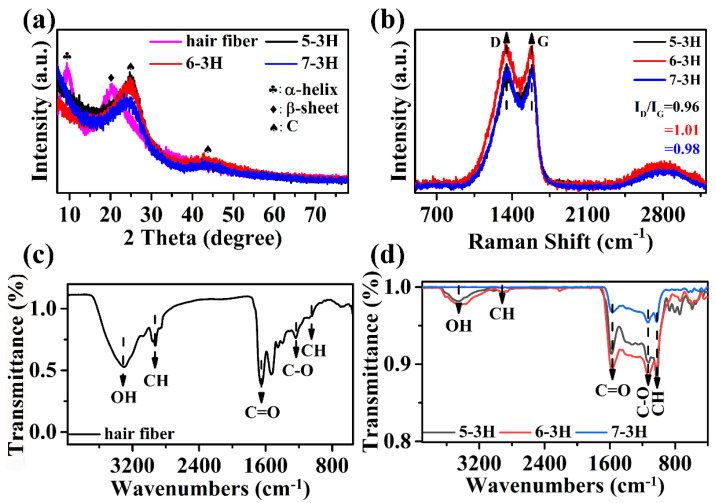
(**a**) XRD patterns of hair, 5−3H, 6−3H, and 7−3H; (**b**) Raman spectra of 5−3H, 6−3H, and 7−3H; (**c**) FT−IR spectrum of hair; (**d**) 5−3H, 6−3H, and 7−3H.

Figure 2 shows the SEM images of 5−3H, 6−3H, and 7−3H. As shown in Figure 2a–c, 5−3H is the sheet structure with a thickness of about 3 μm. Both 6−3H (Figure 2d–f) and 7−3H (Figure 2g–i) are also sheet structures with a diameter of about 3 μm, and their surfaces are smooth. According to the SEM images, 5−3H, 6−3H, and 7−3H do not have the abundant pore structure of activated carbon, which indicates that it is not activated carbon. As shown in Figure 2j–p, 6−3H uniformly contained C, N, S, O, and P elements and a small amount of Cl element. This indicates that the hair-based carbon sheets prepared by the carbonization method can realize the co−doping of many elements.

XRD analysis was performed in order to study the structure of samples. In Figure 3a, the hair exhibited two peaks at around 9.9° and 21.2°, corresponding to the α-helix and β-sheet structure of the protein, respectively [26]. In Figure 3a, for the 5−3H, 6−3H, and 7−3H samples, it can be seen that there are two peaks at 25.1° and 44.2° belonging to (002) and (100) crystal planes of graphite, indicating that the carbonization successfully converts the hair into carbon materials [27]. The structure of the sample was analyzed by Raman spectroscopy using G (typical graphitized sp^2^ C−C bond vibrations) and D (structural defects caused by carbon disorder) vibration bands. The intensity ratio (I_D_/I_G_) of D−band and G−band can reflect the order of graphite [26]. The larger the I_D_/I_G_, the higher the degree of defects in the carbon material. It can be seen that the samples of 5−3H, 6−3H, and 7−3H have D band and G band at 1346 and 1580 cm^−1^, respectively. It can be seen from Figure 3b that with the increase in carbonization temperature, the I_D_/I_G_ values of 5−3H, 6−3H, and 7−3H were 0.96, 1.01, and 0.98, respectively. This is more clearly proven by the area ratio (A_D_/A_G_) of peak separation processing, and the A_D_/A_G_ values of 5−3H, 6−3H, and 7−3H were 2.72, 3.65, and 2.84, respectively (Figure S2). It can be seen that the I_D_/I_G_ or A_D_/A_G_ value of 6−3H was the largest, which means that the 6−3H has the highest degree of defects. As shown by the FTIR spectrum in Figure 3c, the hair showed the strong characteristic peaks at 3420 cm^−1^ (O−H stretching) [27], 1670 cm^−1^ (C=O bending), and 2991 cm^−1^ (C−H bending). As shown in Figure 3d, the FTIR spectra of 5−3H, 6−3H, and 7−3H display the characteristic peaks of C−H, C−O, C=O, C−H, and OH bonds at 903, 1063, 1592, 2856, and 3439 cm^−1^, respectively [28]. It should be pointed out that the intensity of the infrared characteristic peak of samples after carbonization decreases obviously compared with the hair (Figure 3c,d), and the intensity of the characteristic peak decreases gradually with the increase in carbonization temperature. This indicates that the carbonization temperature has an important effect on the structure and surface functional groups of the BCMs, which is consistent with the previous research results on BCMs [11].

### 3.2. Gas sensing Performances of 5−3H, 6−3H, and 7−3H

**Figure 4:**
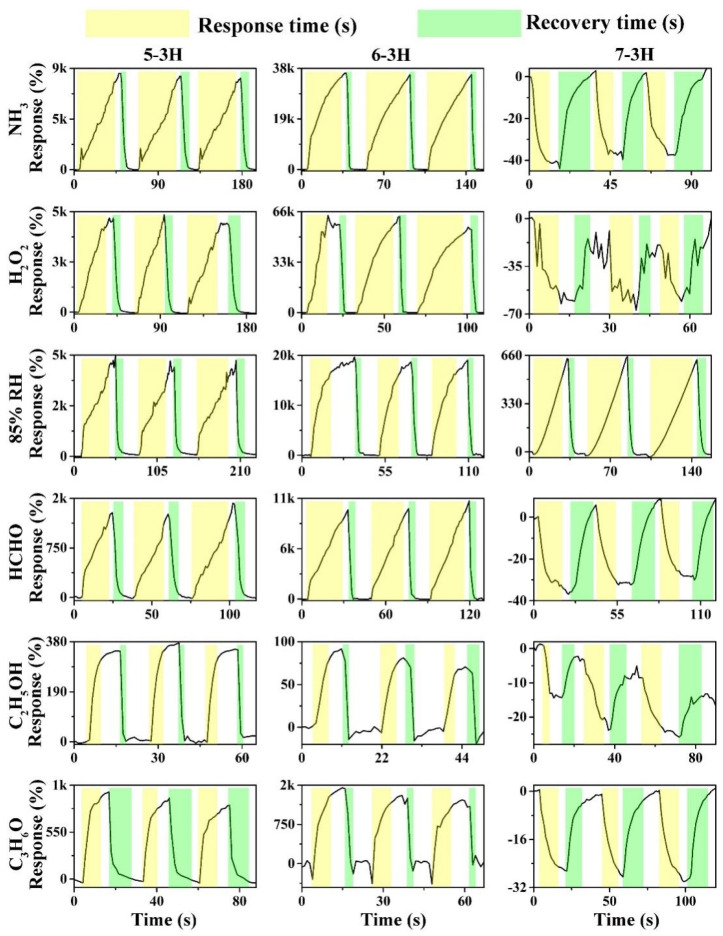
Dynamic sensing curves of the devices based on 5−3H, 6−3H, and 7−3H to 1000 ppm of NH_3_, H_2_O_2_, C_3_H_6_O, CH_2_O, and C_2_H_5_OH vapors and 85% RH at room temperature.

As shown in Figure 4, the hair-based 5−3H, 6−3H, and 7−3H displayed good recoverability in three continuous sensing intervals to 85% relative humidity (RH) and 1000 ppm of NH_3_, H_2_O_2_, C_2_H_5_OH, C_3_H_6_O, and HCHO. On the whole, the gas sensing properties of carbon materials prepared at different temperatures have changed significantly. The responses of 5–3H material to NH_3_, H_2_O_2_, 85% RH, HCHO, C_3_H_6_O, and C_2_H_5_OH were 8233.6, 4890.3, 4342.4, 1317.16, 369.3, and 216%, respectively. This reflects that the 5−3H material is more sensitive to NH_3_, H_2_O_2_, and 85% RH than HCHO, C_3_H_6_O, and C_2_H_5_OH. 6−3H has a similar trend to 5−3H, but the sensitivity of 6−3H has been significantly improved. The responses of 6−3H to NH_3_, H_2_O_2_, 85% RH, HCHO, C_3_H_6_O, and C_2_H_5_OH were 35,724.6, 60,469.3, 18,949.3, 20.7, 1323.7, and 30.1%, respectively. Interestingly, the 7−3H material shows completely different sensing characteristics from the 5−3H and 6−3H. The responses of 7−3H to NH_3_, H_2_O_2_, 85% RH, HCHO, C_3_H_6_O, and C_2_H_5_OH were 38.6, −61.2, 645.3, −32.6, −21.5, and −27.6%, respectively. Not only did the responses of the 7−3H decrease greatly, but their responses are also basically negative. This may mean that the high-temperature carbonization at 700 °C changes the semiconductor type of the hair-based carbon sheets.

**Figure 5:**
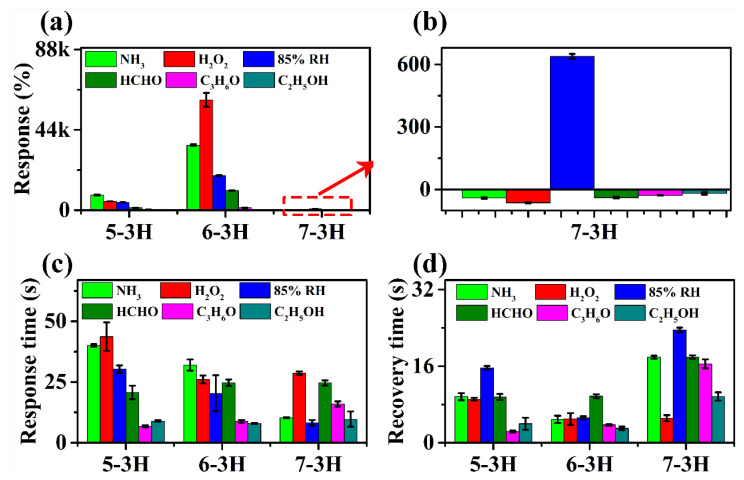
(**a**) Response, the red arrow indicates that (**b**) is a magnified part in the red dotted box in (**a**); (**b**) enlarged part of (**a**); (**c**) response time; (**d**) recovery time of 5−3H, 6−3H, and 7−3H over 3 peaks for 1000 ppm of NH_3_, H_2_O_2_, C_3_H_6_O, CH_2_O, and C_2_H_5_OH vapors and 85% RH at room temperature.

As shown in Figure 5a, with the increase in carbonization temperature, the sensing performance of hair-based carbon sheets first increases and then decreases on the whole. Among 5−3H, 6−3H, and 7−3H, 6−3H was the most sensitive and had the largest response to H_2_O_2_ among the six analytes. H_2_O_2_ is one of the raw materials for making high-explosive triacetone triperoxide [29]. Therefore, the effective detection of H_2_O_2_ is an important means to monitor high-explosive triacetone triperoxide [30]. Interestingly, the responses of 7−3H to the target vapors and gases are not only greatly reduced, but also become negative, except for the 85% RH. This may be because the high temperature (700 °C) changes the microstructure of the 7−3H, transforming the carbon sheet into a P−type semiconductor. The response time of samples prepared at different temperatures to the target gases has no obvious change trend. For some gases, the response time is shortened, while for others, the corresponding time does not change significantly. As for the recovery time, the recovery time of samples to the target gases basically shows a trend of decreasing first and then increasing. These phenomena show that the carbonization temperature has an obvious influence on the structure and gas sensing performance of the samples. However, on the whole, the sensor of the carbon sheet does not show good selectivity.

**Figure 6:**
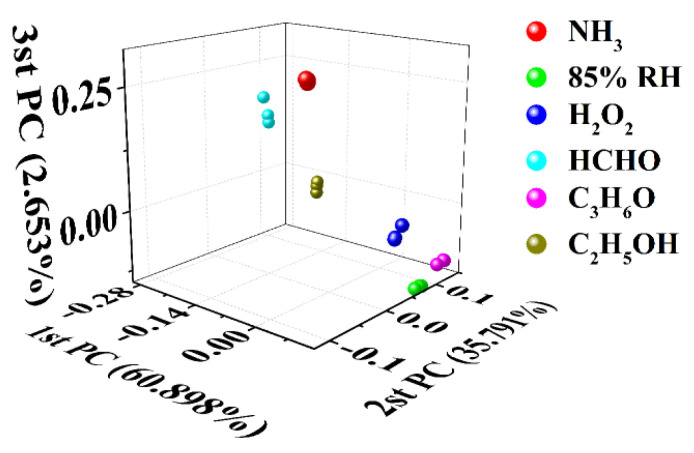
Principal component analysis diagram derived from the responses of 5−3H, 6−3H, and 7−3H to 1000 ppm of NH_3_, H_2_O_2_, C_3_H_6_O, CH_2_O, C_2_H_5_OH vapors and 85% RH at room temperature.

Due to the effect of carbonization temperature on the microstructure of the samples, the samples prepared at different temperatures have different responses to the target gases. This may indicate that it is a simple and feasible method to regulate the gas−sensitive performance of hair-based carbon sheets by changing the carbonization temperature, and then construct the gas sensor array. Therefore, in order to preliminarily evaluate its potential for building a sensor array, a virtual sensor array, including 5−3H, 6−3H, and 7−3H, was built according to the previous test results in Figure 4. Concretely, three continuous responses of each sensor to six target gases were collected and analyzed with principal component analysis, and the discriminative effect of the virtual sensor array was evaluated. As shown in Figure 6, the responses of 5−3H, 6−3H, and 7−3H to target gases were used for principal component analysis. As shown in Figure 6, data points corresponding to the same analyte are clustered together, while data points corresponding to different analytes are distributed in different places and can be distinguished. This shows that six target gases can be discriminatively detected only by a sensor array consisting of three sensors prepared by changing the carbonization temperature.

### 3.3. Limit of Detection and Possible Sensing Mechanisms of WCF−MoS_2_

**Figure 7:**
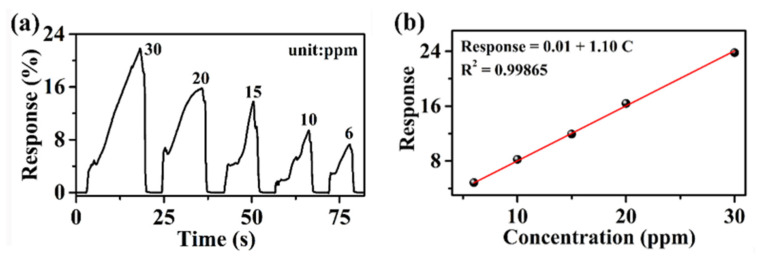
(**a**) Response curves of the sensor 6−3H to different concentrations of H_2_O_2_; (**b**) the fitting plots of response Vs concentration of H_2_O_2_.

Limit of detection (LoD) is one of the most important parameters of a gas sensor [11]. Since the 6−3H has the highest response to H_2_O_2_, the LoD of the 6−3H sensor for H_2_O_2_ was estimated. As shown in Figure 7a,b, different concentrations of H_2_O_2_ were detected by the 6−3H sensor and the responses increased almost linearly with the increase in H_2_O_2_ concentration. According to LoD = 3S_D_/m, the LoD of the 6−3H sensor was calculated to be about 0.83 ppm, which indicates that the 6−3H sensor has a high sensitivity to H_2_O_2_. 

In order to comprehensively evaluate the gas sensing performance of 6−3H, recent H_2_O_2_ sensors are compared in the form of Table 1. In terms of response time and recovery time, the 6−3H sample is at the same level as MoS_2_/RGO−3 [30], CQDs/PCFT [31], and CoPc-f-MWNTs [32], and is superior to SWCNTs [33], MWCNTs/SnO_2_ [34], and Pt-SWCNTs [35]. However, the LoD of the 6−3H sample is only lower than that of MoS_2_/RGO-3 [30], CQDs/PCFT [31], and Pt-SWCNTs [35]. This may be because SWCNTs are loaded with precious metal Pt nanoparticles, CQDs are loaded on fiber membranes with good permeability, and RGO and MoS_2_ form a heterojunction. This shows that the gas-sensitive performance of 6-3H derived from hair for H_2_O_2_ is almost comparable to star nanomaterials such as carbon nanotubes, graphene, and CQDs. Perhaps, the gas sensing performance of 6-3H sample can be further improved by loading precious metals or building heterojunctions.

**Figure 8:**
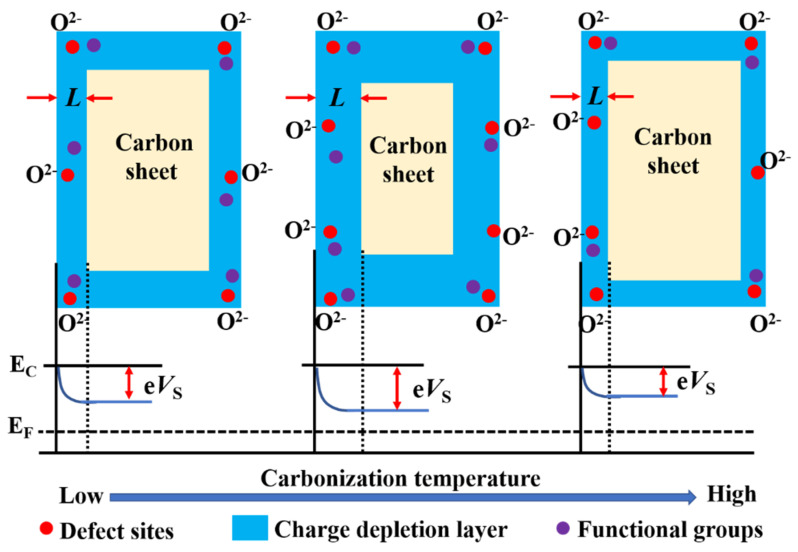
Possible sensing mechanisms of the 6−3H sample.

The sensing mechanism of chemiresistive gas sensor for H_2_O_2_ may be explained as follows. Firstly, when the sensing material is exposed to air at room temperature, oxygen will be adsorbed to the sensing material (Figure 8), according to Equations (3) and (4).

O_2_ (gas) → O_2_ (ads) (3)

(4) $$O_{2}\;(\mathrm{ads})+e^{-}\to O_{2}^{-}\;(\mathrm{ads})$$

The existing state of H_2_O_2_ and the reaction with the sensing material mainly depend on the concentration of H_2_O_2_. At high (>10 vol%) concentrations, H_2_O_2_ exists according to Equation (5) [35].

2H_2_O_2_ = 2H_2_O + O_2_ (5)

At lower concentrations (<2.1 vol%), H_2_O_2_ exists according to Equation (6) [36]:

2H_2_O_2_ = 2H_2_O + 0.87O_2_ + 0.08O_3_ (6)

In this work, H_2_O_2_ with a mass fraction of 30% was used and the main product of H_2_O_2_ decomposition should be H_2_O and O_2_ according to Equation (5). Therefore, the O_2_ generated by the decomposition of H_2_O_2_ is adsorbed to the sensing material, such as the O_2_, in the air to capture more electrons from the sensing material [30]. In this way, the charge depletion layer (*L*) of the sensing material will be larger, so the sensing sensitivity will be higher (Figure 8). Almost at the same time, the sensor will make contact with the H_2_O vapor generated by the decomposition of H_2_O_2_ and have a greater response to the H_2_O vapor, which is the special sensing signal of H_2_O_2_ [30].

Secondly, the carbonization temperature also has an obvious effect on the gas sensing performance of the hair-based carbon sheet [37]. Different carbonation temperatures lead to different structural parameters of samples, such as the conductivity, defect sites, functional groups, etc., which jointly affect the gas sensing performance of sensing materials [11,16]. Generally speaking, the larger the *L* of the sensing material, the higher the sensitivity when the particle size of the sensing material is constant [10,38]. The *L* is directly proportional to the oxygen ion concentration on the surface of the sensing material and inversely proportional to the carrier concentration [31,39]. According to the FTIR and Raman spectrum, 6−3H has the most functional groups and defects among the 5−3H, 6−3H, and 7−3H samples, which is conducive to the adsorption of oxygen and the improvement of *L*. What is more, the content of impurity atoms has a significant impact on the gas sensing properties of semiconductor materials and the optimum effect can be achieved only with appropriate doping amount [15,16]. According to the literature, with the increase in carbonization temperature, the impurity atoms (such as N, O, and S) in the hair-based carbon sheet are gradually reduced (Table S1) [37]. Compared with the atomic doping too high in 5−3H and too low in 7−3H, the atomic doping in 6−3H is moderate, so it also has better gas sensing performance.

At last, with the increase in carbonization temperature, the conductivity and carrier concentration of the sensing material also increase, which is not conducive to the improvement of *L* [11]. Therefore, in terms of gas sensing performance, the carrier concentration has a competitive relationship with the functional groups, defects, and impurity atoms of the sensing material. It may be that the competition of the above comprehensive factors leads to the 6−3H with the largest *L* and the highest sensitivity.

## 4. Conclusions

Waste hairs were carbonized into carbon sheets doped with K, N, P, and S elements and studied as gas sensing materials for the first time. The structure and gas sensing properties of the hair-based carbon sheet were successfully tailored by controlling the carbonization temperature. Although the carbon sheets prepared at different temperatures have different responses to the target gases, the anti-interference ability of the single sensor based on carbon sheet to the target gases was insufficient. Fortunately, the virtual sensor array constructed by 5−3H, 6−3H, and 7−3H prepared at three different temperatures realized the discriminative detection of six gases. This study provides a reference for the simple and low−cost preparation of gas sensing materials and the effective regulation of gas sensing properties.

## Figures and Tables

**Table 1:** Sensing performance of the recently reported H_2_O_2_ gas sensors.

Sensing Methods	Concentration (ppm)	Response (%)	Res/Rec Time (s)	LoD (ppm)	Ref
MoS_2_/RGO−3	50	373	8.7/2.1	0.65	[30]
CQDs/PCFT	100	12,000	10/2.5	0.057	[31]
CoPc−f−MWNTs	34% H_2_O_2_ (aq) vapor	3.8	2/3	--	[32]
SWCNTs	100	~1.97	~20/18	25	[33]
MWCNTs/SnO_2_	17.5	~59,500	210/342	--	[34]
Pt−SWCNTs	60.6	50	~240/--	0.027	[35]
6−3H	6	760	8.6/2	0.83	Present work

## Data Availability

The data presented in this study are available on request from the corresponding author.

## Supplementary Materials

The following supporting information can be downloaded at: https://www.mdpi.com/article/10.3390/nano12193512/s1, Figure S1: Schematic process of gas sensing test; Figure S2: Raman spectra of (a) 5−3H, (b) 6−3H and (c) 7−3H and area ratio (AD/AG) according to peak separation processing; Table S1: Chemical composition of HMC materials determined by energy-dispersive X-ray spectroscopy (EDX) measurements and X-ray photoelectron spectroscopy (XPS) [37].
