# Supplementary material for: Preparation and Gas Sensing Properties of Hair-Based Carbon Sheets
Source: Nanomaterials (Basel). 2022 Oct 8;12(19):3512. doi: 10.3390/nano12193512 (PMC9565493; doi:10.3390/nano12193512)
Supplement: Supplementary file 1 [file nanomaterials-12-03512-s001.zip › nanomaterials-1896741-supplementary.pdf]

nanomaterials

Supplementary Materials

Preparation and Gas Sensing Properties of Hair-Based Carbon Sheets

Zhaofeng Wu [1,2], Yidan Xia [1,2], Lixiang Liu [1,2], Qihua Sun [1,2], Jun Sun [1,2], Furu Zhong [3], Min Zhang [1,2,*], and Haiming Duan [1,2,*]

1 Xinjiang Key Laboratory of Solid State Physics and Devices, Urumqi 830046, China
2 School of Physics Science and Technology, Xinjiang University, Urumqi 830046, China
3 School of Physics and Electronic Science, Zunyi Normal College, Zunyi 563006, China
* Correspondence: minzhang0816@163.com (M.Z.); dhm@xju.edu.cn (H.D.)

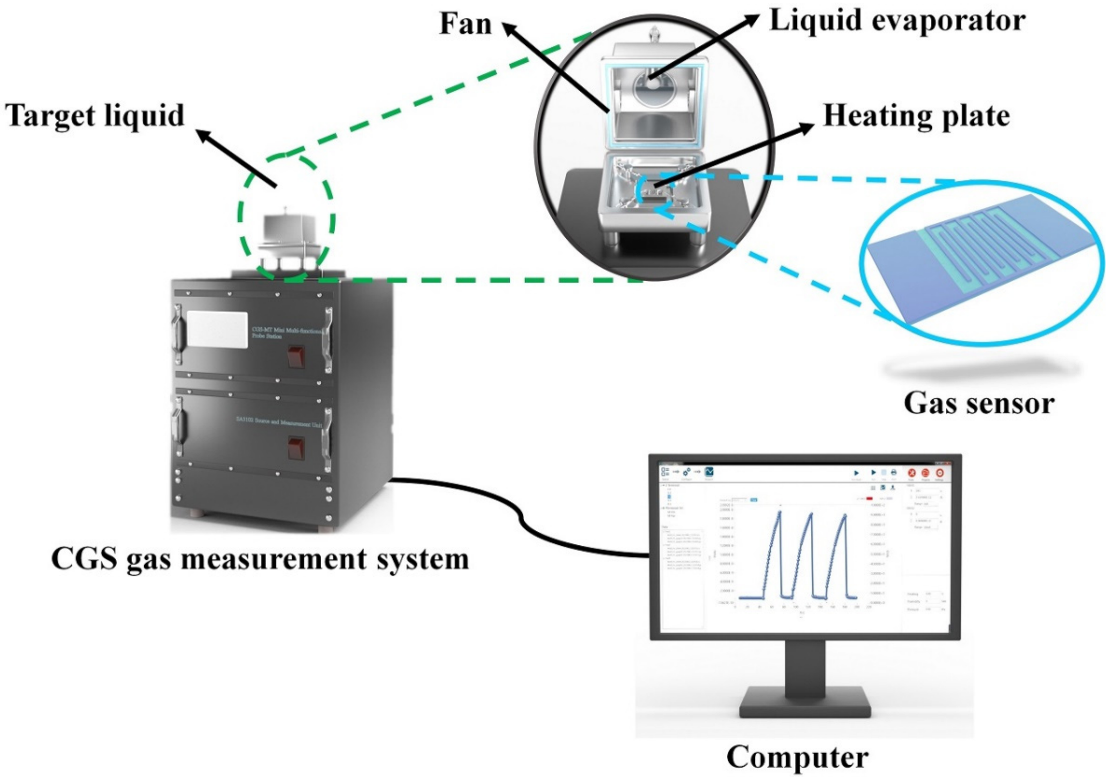

Figure S1. Schematic process of gas sensing test.

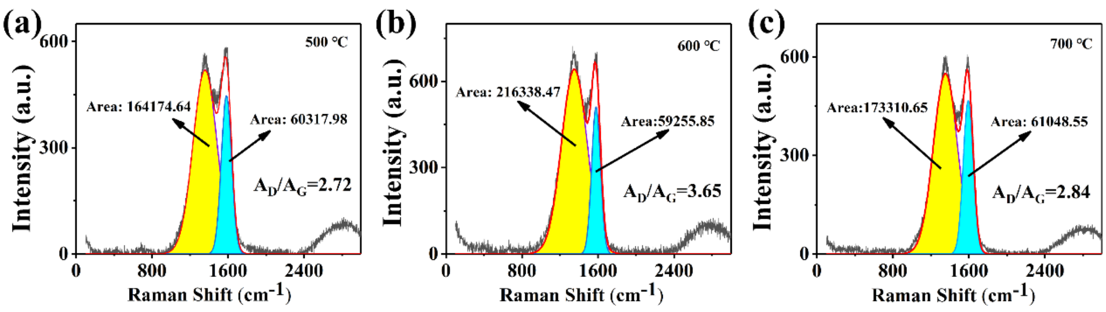

Figure S2. Raman spectra of (**a**) 5-3H, (**b**) 6-3H and (**c**) 7-3H and area ratio (A_D/A_G) according to peak separation processing.

Table S1. Chemical composition of HMC materials determined by energy-dispersive X-ray spectroscopy (EDX) measurements and X-ray photoelectron spectroscopy (XPS) [1].

	EDX (wt%)				XPS (atom%)			
	C	N	O	S	C	N	O	S
HMC-700	82.90	5.62	8.99	2.49	85.20	5.12	7.45	2.23
HMC-700	88.20	4.74	5.53	1.54	88.72	4.38	5.39	1.51
HMC-700	91.32	2.33	4.98	1.37	92.30	1.96	4.28	1.46

References

1. Qian, W.; Sun, F.; Xu, Y.; Qiu, L.; Liu, C.; Wang, S.; Yan, F. Human hair-derived carbon flakes for electrochemical supercapacitors. *Energy Environ. Sci.* **2014**, *7*, 379–386. https://doi.org/10.1039/C3EE43111H.

Special note: This table refers to the contents in Reference [1].
